# Recent Progress on Lignocellulosic-Based Polymeric Materials

**DOI:** 10.3390/polym18121498

**Published:** 2026-06-15

**Authors:** Adrian Cătălin Puițel, Mircea Teodor Nechita

**Affiliations:** Faculty of Chemical Engineering and Environmental Protection “Cristofor Simionescu”, Technical University “Gheorghe Asachi” Iasi, Bd. Prof. Dimitrie Mangeron, No. 73, 700050 Iaşi, Romania; adrian-catalin.puitel@academic.tuiasi.ro

Lignocellulosic biomass has long been recognized as the most abundant renewable source of organic matter on Earth. Primarily composed of cellulose, hemicellulose, and lignin [1], lignocellulosic materials may be defined as products resulting from biomass processing that preserve attributes such as sustainability and eco-friendliness while aiming to replace traditional petrol-based products in many applications [2]. Switching to lignocellulosic-based polymeric materials is primarily motivated by the need to transition from fossil carbon-based resources to sustainable, circular, bio-based, and sometimes economically cheaper, alternatives [3].

The transition may be sped up by environmental regulations, increasing knowledge of processing technologies, and rising educated consumer demand for eco-friendly products [4]. On the other hand, biomass recalcitrance is the main reason why the processing and valorization of various products may be regarded as challenging. Continuous research efforts are targeting the particular, specific, and optimal conditions of raw material components’ separation and further value-adding to all separated components [5].

Lignocellulosic-based polymeric materials represent the primary renewable resource for bio-based materials and chemicals, as well as being promising, efficient, and sustainable solutions for environmental remediation. Oxidation of cellulose microcrystalline powder resulted in new aerogel-type materials that can be used for the decontamination of marine sediments [6]. Assessing the environmental performance of the studied materials led to conclusions regarding the best synthesis route, as well as the associated environmental impact.

Biomass polymeric composition can influence the results of thermochemical processing. Armanu and coworkers provided an assessment of the main characteristics of three different feedstocks and of the relations between biomass polymeric composition, thermochemical conversion path, and char yield [7]. Thus, the authors demonstrated that the char yield can be predicted and is strongly dependent on the biomass polymeric composition.

Agricultural residues or agro-industrial byproducts are well known as a viable feedstock for the production of biopolymers [8], with the potential to replace wood as a cheaper source of cellulose for further chemical modification [9]. The work of Nakyp et al. details the process of cellulose extraction from rice straw using water–alkaline solution treatment and the subsequent process of carboxymethylation of the obtained product when activated by microwave radiation [10]. The study is particularly important because of the high availability of rice straw as an agricultural byproducts. Unlike conventional strategies for chemical modification of cellulose, etherification under microwave irradiation offers the possibility of obtaining cellulose derivatives in a faster manner and with better values of degree of substitution [11].

The paper concerning the potential use of Medicago sativa stems as a feedstock in a multi-output integrated biorefinery approach [12] falls under the same umbrella as the previous discussion: the valorization of agri-wastes. The work addresses the case of potentially lost lucerne stems as a result of improper forage management. By using the stems to obtain protein-rich extracts and further processing them to create papermaking fiber, hemicelluloses, and lignin-rich fractions, waste lucerne stems reveal their potential as a new raw material.

The discussion concerning the integrated biorefinery concept is to be continued if the subject of lignin valorization is taken into account. The work of Driscoll et al. covers the production of functional materials from lignocellulosic biomass [13]. In this proof-of-concept study, lignin-derived, fully bio-based polyesters were synthesized via a simple metal-free, organo-catalytic method as a way to reduce the environmental strain and replace fossil-based polymers and plastics.

Production of bacterial cellulose is of particular importance because of its high-value polymer characteristic, with a wide range of potential applications in multiple industry sectors [14]. Quiñones-Cerna et al.’s study addresses optimization using artichoke bract as a culture medium for bacterial cellulose production [15]. The findings validate the technical and structural feasibility of the produced biopolymer and highlight the valorization potential of a low-cost agricultural residue through an eco-efficient biotechnological process, contributing to the development of functional materials derived from renewable sources.

The importance of hydrogen bonds in lignocellulosic biomass and lignocellulosic polymeric material structures has long been acknowledged by the scientific community [16,17]. Hydrogen bonding operates both intra- and intermolecularly, as well as through matrix type bonding, with hemicelluloses acting as a natural binder of lignin and cellulose [18]. Hydrogen bonding is responsible for both lignocellulosic biomass and the resulting processing products’ structural integrity, mechanical strength, and even resistance to chemical or biological degradation [19].

The modification of hydrogen bonds in different types of wood species can enhance existing properties or induce new ones. Generally, whether organic or inorganic, most of these treatments target mechanical properties, dimensional stability, and durability, leading to better utilization of some wood species, and improving the ecofriendliness of many applications [20]. The importance of wood treatment for increased stability and durability is also a research objective for Andze and coworkers, who show that sodium hydroxide treatment alone results in increased mechanical performance compared with more complex chemical routes, while relying on milder chemistry and preserving the bulk polymer framework of the wood [21].

Finally, lignocellulosic biomass and derived polymeric materials continue to establish themselves as a foundation of sustainable materials science, offering versatile pathways toward the replacement of fossil-based resources across multiple industrial sectors. The research presented in this Special Issue highlights the possibilities arising from the valorization of cellulose, hemicellulose, and lignin—from aerogel-type decontamination materials and bio-based polyesters to bacterial cellulose and chemically modified agricultural residues. The integrated biorefinery concept, supported by advances in thermochemical processing, chemical modification, and biotechnological approaches, proves central to revealing the full potential of both dedicated biomass crops and agro-industrial byproducts. Furthermore, a deeper understanding of structural features, such as hydrogen bonding, alongside optimized treatment strategies, continues to drive improvements in the mechanical performance and durability of lignocellulosic products. Collectively, these efforts reflect a growing scientific commitment to advancing lignocellulosic-based polymeric materials as sustainable, circular, and economically viable solutions for the challenges of a post-fossil fuel economy.
